# Making people meet: volunteers’ contributions to social connection for the well-being of self and others

**DOI:** 10.1080/17482631.2025.2476215

**Published:** 2025-03-07

**Authors:** Nina Petersen Reed, Julia Hagen

**Affiliations:** a Department of Mental Health, Norwegian University of Science and Technology, Trondheim, Norway; b Department of Mental health work, NTNU Samfunnsforskning AS (NTNU Social Research), Trondheim, Norway

**Keywords:** Social connection, well-being, relationships, volunteering, activity, mental health

## Abstract

**Purpose:**

Social isolation and loneliness are significant public health challenges that reduce well-being. The voluntary sector is suggested to be an important contributor in reducing loneliness and enhancing social connectedness and well-being in everyday life. This study aimed to contribute knowledge about how volunteers may help promote social connections and well-being, by exploring the experiences and perspectives of volunteers themselves.

**Methods:**

We conducted a qualitative study in Norway interviewing nine volunteers. Data was analysed using reflexive Thematic Analysis.

**Findings:**

We developed three themes: *Being part of and facilitating a variety of meaningful relationships and roles, Creating spaces of trust in places of shared activity*, and *Calling for more diversity in volunteering while acknowledging challenges*.

**Conclusions:**

Volunteers’ may contribute to social connection and well-being, particularly through arranging social activities where relationships are equal and established spontaneously. Some challenges remain before volunteer activities are fully inclusive for all. Extended organizational support of volunteers may be necessary for recruitment and continuity of volunteer efforts towards some marginalized groups.

## Introduction

Social connections and a sense of community where people feel valued are fundamental to people’s health and well-being (Baumeister & Leary, 1995; Holt-Lunstad, 2022; Prilleltensky, 2020), while social isolation and loneliness are associated with ill physical and mental health (Leigh-Hunt et al., 2017). Social connection is an umbrella term for the structure, function, and quality of a person’s social relationships. The structure of social connections may be depicted based on their interactions and roles in an individual’s life and quantified as social networks. The function and quality of social connections encompass for instance affective components, perceived social support, reciprocity, and the meaningfulness of the different relationships in an individual’s social network (Holt-Lunstad, 2022). Mattering is also developed through social connections and can be described as feeling valued and adding value to self and others in the community (Prilleltensky, 2020). According to Prilleltensky, there is a need to balance feeling valued with adding value, as well as to balance adding value to self with adding value to others. In that sense, mattering contributes to well-being, not only on a personal level but also on a relational and collective level. Furthermore, a balance in feeling value and adding value, to self and others, contributes to a “we culture” where experiences of well-being and fairness are sought for all (Prilleltensky, 2020).

Unfortunately, not all individuals experience adequate social connections, and loneliness is a major public health issue (Leigh-Hunt et al., 2017; Surkalim et al., 2022). Developing and maintaining social connections may be particularly challenging for marginalized groups such as individuals with poor physical or mental health or substance use problems, as well as individuals with inadequate financial resources. Therefore, they may face greater loneliness and social isolation than the rest of the population (O’sullivan et al., 2021; Vigdal et al., 2023; Wilkinson et al., 2019). Promoting social connections and preventing and reducing loneliness should therefore be a focus for public health measures, particularly for marginalized groups (Holt-Lunstad, 2022; Leigh-Hunt et al., 2017; Ministry of Health and Care Services, 2015; Surkalim et al., 2022). The WHO also emphasizes that supportive environments and community action are important in health promotion as health is created in individuals’ everyday lives in the arenas where they live and act (Von Heimburg & Ness, 2021; World Health Organization, 1986). Relationships that contribute positively to social connections may be developed via participation in social activities within everyday life contexts, such as family events, work, leisure activities, or talking with neighbours, staff at a grocery store, or others in the local community (Wilkinson et al., 2019).

According to the Norwegian Ministry of Health and Care Services (2019) we need more knowledge about public health measures that might prevent loneliness and provide social support. The voluntary sector has been suggested as an important contributor to enhance social connectedness and reduce loneliness together with the public sector (Leigh-Hunt et al., 2017; Ministry of Health and Care Services, 2015). Volunteering may be defined as “activities that are chosen freely, done without monetary recompense and carried out without fear of sanctions if one withdraws” (Henriksen et al., 2019, p. 8). Hustinx and Lammertyn (2003) describe two styles of volunteering, the collective and the reflexive. Collective volunteers are motivated by belonging, loyalty and duties towards groups, often tied to religious or altruistic values. Reflexive volunteers are motivated by individual preferences and interests, however often together with altruistic values. Hustinx and Lammertyn (2003) further write that a reflexive style of volunteering becomes more common as societies develop in individualized directions, and that organizations may have to adapt to more interest-driven and ad hoc contributions by volunteers. Studies on volunteering in a Norwegian context show that reflexive volunteering, where volunteers are motivated for the good of both themselves and others, is indeed most common (Hansen & Slagsvold, 2020; Onstad et al., 2021).

Norwegian authorities argue that volunteer organizations may play an important role in preventing loneliness by creating inclusive local communities and meeting places, and by organizing activities which may promote social connections between individuals or in groups. They uphold that the volunteer sector can reach citizens that the public sector cannot (Ministry of Health and Care Services, 2015). Previous research has shown that for volunteers themselves volunteering may indeed provide valuable contributions to increase social interactions and reduce loneliness (Carlà et al., 2023; Vannier et al., 2021; Williams et al., 2022), provide individual social capital through social networks (Mutz et al., 2022), as well as to enhance social connectedness and well-being (Brown et al., 2012). However, there is limited research on how volunteer efforts can promote social connections and reduce social isolation and loneliness for others (Gadbois et al., 2024; Williams et al., 2022). There is some evidence that volunteers can be an important resource of social connection for marginalized groups such as homebound older adults (Gadbois et al., 2024). For individuals with mental health or substance use problems, volunteers may enhance capacity and self-esteem for socializing, and function as stepping stones to socialization and networking (Sheridan et al., 2018). Further, volunteer “befriending” may provide much needed non-professional relationships for these groups, that contribute well-being and change (Mitchell & Pistrang, 2011), as well as increased social contact and social confidence (Cassidy et al., 2019; Priebe et al., 2020). The research reviewed here suggests volunteers may be a resource for social connection for others, and in particular for marginalized groups. However, there seems to be a lack of research about how volunteers think and act to acquire this.

The purpose of this study was to contribute knowledge about how volunteers may help promote social connections and well-being. We chose to do so by exploring the experiences and perspectives of volunteers themselves. Our research question was: How do volunteers understand their role in promoting social connections and well-being between fellow citizens through shared activities?

## Methods

This study is based on an explorative and qualitative design, and we generated data through semi-structured individual interviews and analysed the material using reflexive Thematic analysis (Braun & Clarke, 2022). Qualitative methods are suitable for gaining insights into people’s experiences and perspectives, and the context in which they live and interact (Malterud, 2017). We conducted the study from a social constructionist standpoint, viewing knowledge as co-constructed, contextual, and changeable (Gergen, 2015).

### Recruitment and participants

Participants were recruited through purposeful sampling and snowball recruitment (Patton, 1990). First, we sent information about the study as well as a request for permission and help to recruit volunteers to two formal organizations offering social activities through volunteer efforts, both located in the city of Trondheim, Norway. One is a relatively small association primarily directed towards individuals with mental health and substance use problems and their next of kin. The other was Red Cross, a large international non-governmental organization with offers directed to a variety of target groups such as children, families, immigrants and elderly. Both organizations forwarded our requests to their volunteers, and three and four participants from each of them contacted us willing to participate in the study. One of the organizations also forwarded our request to another volunteer group, an informal hiking group organized through Facebook aimed towards individuals who just wish for someone to walk and talk with. This resulted in two additional participants.

Four of the volunteers were men, and five were women, ranging from the mid-20s to the early 70s in age. Although recruited from only three different organizations/groups of volunteers, they were engaged in several activity offers in these, as well as in other organizations. Five participants mainly arranged two different hiking groups and bonfire- talks in the local forest. The four other participants were hosts at local community cafes open to all citizens who wish to come during daytime/working hours, engaged in individual home visits or one-to-one activities with elderly or individuals with mental health problems, or contributed at volunteer centres offering practical help for individuals in their local community such as refugees and elderly. Three of the volunteers shared that they had experienced mental health problems and loneliness, having contacted volunteer groups on that ground. Thus, these three spoke from the perspective of being participants in activities organized by volunteers, as well as from the perspective of being volunteers themselves.

### Data generation

The first author conducted individual, semi-structured interviews with all nine participants. Eight interviews were conducted in a meeting room at the first author’s workplace. One interview was conducted at a café, upon participant request. The interviews lasted between 23–60 min, with an average of 34 min. The participants were asked to talk about their volunteer efforts, and how they wished their efforts would contribute to others’ everyday lives, relationships, and social networks. They were also asked what they do to facilitate relationships while volunteering.

### Data analysis

We analysed the data using reflexive Thematic Analysis (TA), which provides a systematic yet flexible framework for identifying, analysing, and reporting patterns (themes) of meaning across qualitative data (Braun & Clarke, 2022). Our inductive analysis stayed close to the surface of the data, performing TA at a semantic level. Both authors have a professional background within practice and research on mental health, and particularly the first author’s previous work on meaning-making through shared activities inspired this project and our analysis and interpretations of the participants’ experiences. We collaborated in interpreting the data and conducted the analysis in the following (simplified) steps: 1) We read and re-read the transcripts to get familiar with the content, noticing relevant parts of the data and scribbling notes in the margins. 2) We shared the material between us, creating a document with the relevant segments of the coded data. The first author continued to work with the whole material, and we discussed the coding process and code labels in several meetings. 3) The first author developed initial themes by combining codes that fit together. 4) During this process, we met several times to discuss the initial themes and further develop our interpretations. We moved back and forth between the transcripts, coded materials, and initial themes. 5) We discussed, refined, and agreed on the final themes and analytic narratives. See Table I for a simplified example of the analysis.

**Table I.** Example of analysis.

Statement	Initial coding	Initial theme	Final theme
“It is much easier to talk in nature rather than sitting face to face”	Easier to talk out in nature.	Being outside and in activity makes it easier to talk with people.	Creating spaces of trust in places of shared activity
“So we have a common interest, right? We love hiking.”	Important with common interest	Common interests are important to create relationships through activity	
“We are becoming a large group, and that does not fit all.”	Large groups do not fit all.	Individuals have different preferences and needs - there should be something for everyone.	Calling for more diversity in volunteering while acknowledging challenges
“So, I try to keep a bit distanced from it as well. (…) It is quite burdensome to relate to, really … “	Protecting oneself – trying not to get too involved	Volunteering for individuals with mental health problems may be emotionally challenging	

### Ethical considerations

The research project was presented to the Regional Committee for Medical and Health Research Ethics (REK), which determined that it does not fall under their jurisdiction as medical research (reference number 336,121). The project was then submitted to Sikt (Norwegian Agency for Shared Services in Education and Research) for prior assessment. Sikt provided feedback confirming that the planned processing of personal data met the requirements of the EU General Data Protection Regulation (GDPR), (reference number 466,614). The participants were informed that they could withdraw from the study at any time until publication without providing any reason and signed an informed consent form. We treated the data confidentially and present information about the participants and their experiences in such a way that they are not identifiable to others. We refer to the participants by fictitious names and have changed some details about them to protect their anonymity. Additionally, we choose not to include the names of the two smaller volunteer organizations from which we recruited participants, as they were run by only a few volunteers, potentially compromising our participants’ anonymity.

## Findings

The volunteers shared experiences and thoughts on how they may contribute to social connections and well-being among people in several ways through shared activities. Through our analysis, we developed the following three themes: *Being part of and facilitating a variety of meaningful relationships and roles*; C*reating spaces of trust in places of shared activity*; and *Calling for more diversity in volunteering while acknowledging challenges.*

### Being part of and facilitating a variety of meaningful relationships and roles

From what the volunteers told, it seems that they and the individuals participating in the activities they arrange enact several roles and become part of a variety of relationships through their activities. These relationships sometimes appear unclear, and roles may overlap and switch over time.

Several participants said that they started volunteering just as much for their own well-being and social needs as for others, and that they took on the role of volunteers in search of new friendships. Thomas, a volunteer at a local community café and a “visiting friend” expressed that it is quite common for individuals to volunteer based on their own social needs:

I think several volunteers have a social effect from their work anyways. And it is not very common that volunteers come from a completely perfect life to volunteer. That is my experience. So, sometimes it is like … Sometimes it is ok to come to a volunteer organization for new networks for oneself. For new relationships, to meet new people.

This was also illustrated by the group leader of a hiking group, Kirsten: “I started the group both for myself and for others”. She had struggled with anxiety for some time and had to quit her job for a while. She wished for someone to go hiking with, as well as some new friends, and therefore started arranging hiking trips through Facebook. Through these trips, she made new friendships which meant a lot to her.

Kirsten had also observed how the roles in the group develop when individuals participated over time: “Through these hiking trips persons get to mean something for others—the roles in the group develop”. Kirsten further described asking others to take over for her when she could not lead the trips—and how others gladly accepted this. The volunteer Anne similarly observed how roles switch over time: “It is passed on, like rings in water,” referring to how individuals join these groups first to help themselves, and then, when feeling better they become resources for others.

The volunteers’ experiences illustrate people’s relational needs, whether they organize volunteer activities or participate in such activities. For these volunteers, reciprocity between themselves and other participants, shifting between the roles of givers and receivers of support, and forming new, true friendships seemed to be desired. However, Thomas stated that true friendships are not necessarily acquired through volunteering. He had wished to make new friends through volunteer work. However, although he did expand his network and time spent with others, he did not quite find what he was looking for:

When it comes to network … I have met another person to visit once a week. In that way, my network has increased. And that is good in itself, even though it is not real friendship anyways … We have certain defined roles. I am in a way her helper, she is a patient or such. It is not real. It is not balanced on my part, not a real friendship. It is not what I wished for.

Several other volunteers also emphasized that they did not form true friendships with participants. They mentioned different reasons for this, such as not wanting to form true and private friendships, or that they were not allowed to do so by the organization they volunteered for. However, some of them still became close with the person they encountered, as illustrated by Susan who is a “visiting friend” through an organization: “Now I have known her for quite some time, so I know a lot about family, friends and such. Now it feels like … Yes. I would not say quite as friendship, but almost, at least”.

Furthermore, some volunteers explained how individuals they meet need some support during social activities due to mental or physical difficulties. The need for them to provide support made visible unequal roles as volunteers and recipients and could overshadow the more friend-like characteristics of the relationships. Susan, for instance, seemed to struggle with defining her role and relationship with the participant—being neither only a “volunteer”, nor a “personal friend”:

It is a little difficult to be volunteers, because we have a sort of role … And these are things we do for someone else as well. So, it is a little bit … Yes. It is difficult to determine where to draw the line. Between what is volunteer work and what is just friendship.

Further, she thought the person she was a visiting friend for feels like a burden to others because of her difficulties and that she is very conscious of Susan’s role as a volunteer:

I often feel that she thinks I am only with her because it is a voluntary assignment. I think that is a shame, so I try not to focus that much on my role, and rather just be together. I enjoy being with her. It gives me something.

Susan seemed to conceal her role as a volunteer a bit and wished to accentuate a more personal connection. In this way, volunteers’ roles appeared muddled. They did not want to be viewed merely as “volunteer helpers”, and they did not believe that participants wished to be in the role of “recipients of help”. However, they would not identify completely as a “friend” either, even though they might wish to. Even in hiking groups where the roles were mainly perceived as equal and the relationships as friendships, volunteers described some variances in roles related to the volunteers assuming responsibility for the participants, for example by bringing a first aid kit and knowing how to perform first aid. However, Bill seemed hesitant to these kinds of responsibilities: “I do not want it to be that organized”, and followed up by asking: “How serious should this group be?”

The volunteers also facilitated relationship building among the other participants and shared examples of how the same persons participated several times and sometimes made friends with others in the activity, as illustrated by Kirsten:

There are so many nice people. Mostly single people, and they all have a history. I hear that they are so happy for having found this group. They have expanded their network. One, for example, moved to the city and knew no one last year, and now she knows many individuals. She has acquired good girlfriends who are also single. (…) They now go to concerts, movies, trips and travels together.

Thus, some participants of these group activities extended their relationships by making plans to meet and do other activities together on their own initiative. The volunteer Ben had even experienced that two people became a couple, and further indicated that such groups may promote meetings and friendships between people who might not otherwise have met: “There are people you normally would not have met, or considered as new friends, had it not been for this group”. Thus, through group activities arranged by the volunteers where individuals met and interacted with each other, they had experienced that social networks were increased, and establishment of more lasting relationships was made possible. In the following section we show how volunteers actively sought to promote relationships.

### Creating spaces of trust in places of shared activity

The volunteers shared examples of various ways to create spaces of trust where they and others would feel safe to participate and open up to new relationships. These spaces of trust involved safeguarding emotional needs, as well as physical places where the volunteers and participants engaged in activities of common interest.

Several of the volunteers who arranged hiking trips in groups stressed a sensitivity to the emotional needs of participants. They talked about trying to create a positive environment, emotional safety, and openness during the walks. One volunteer, Ben, put it this way: “Primarily, I am there to be social. To build others up. And to spread joy.” However, he also stressed that he wished for the group to be a safe space for sharing problems: “We understand that everyone will have different days, and I think it is important to create relationships that make people feel safe and that are available for them if they have a bad day. That is important”. Being particularly attentive to the newcomers, ensuring they felt welcome in the group and spending time getting to know them was also highlighted by several volunteers. Kirsten said: “Before each hike, I say that we must include all newcomers. They are well taken care of. Yes, because we have all been newcomers.” They explained that managing to show up could be hard for some. Further, Ben stressed that it is important for the volunteers to feel relaxed as well. He explained: “It has something to do with the energy you offer the group. Yes, if you cannot spread joy … If it doesn’t seem like you are … Safety. Trust. Those are the most important words.” They therefore preferred volunteering in activities they had an interest in, where they also felt comfortable and had a good time. Ben highlighted: “So we have a common interest, right? We love hiking. We love going out. We have one big thing in common”. Several of the volunteers similarly suggested that doing activities together and sharing positive experiences facilitated new relationships.

Several volunteers highlighted physical places that they experienced as particularly enabling social interactions. Kirsten, for instance, shared that: “It is much easier to talk in nature rather than sitting face to face, at least so I think. And especially with new individuals which you do not know.” Ben supported this, and additionally suggested that hiking groups in nature might promote health: “To just get out. Just that will promote mental health in abundance. To get out into nature. Share experiences with others. Perhaps start a bonfire and brew coffee on it. Create enjoyment”. Also illustrating the role of physical places and shared activities, Ben described carpooling as a good way of getting to know others:

Sometimes you need to bring people with you in cars, or you must ride with someone else, and then it becomes a small group in these cars sitting there and talking. They have something in common there as well. Yes, they are forced together. (…) And perhaps you discover individuals whom you normally would not contact. Yes, it becomes an icebreaker.

Frank, who had volunteered at a community cafe highlighted these as physical arenas that facilitate contact. He said that these cafés are more than just ordinary cafés serving food and drinks. They are there to create opportunities for physical meetings between individuals in the same neighbourhood who are home during the daytime and perhaps feel lonely. “If you are very ill, this might be the only activity you are able to do during the day. Getting to this place and having a cup of coffee. They are very easily underrated, such meeting places”, Frank said. The volunteers there sought to connect a bit, initiating conversation with all the individuals who came by. Nevertheless, Frank also said that there may be times when these cafés have very few visitors. He explained that the local needs for these offers may vary and that they sometimes moved the café from one area to another. Nuancing this the volunteer Thomas had experienced that individuals may not find personal connections and friendships at these cafés as different people and volunteers show up, and they might have to introduce themselves to new persons each time. However, he underlined: “But coffee meetings were better than nothing.”

### Calling for more diversity in volunteering while acknowledging challenges

The volunteers mentioned several challenges in the voluntary sector which may restrict volunteers’ contributions to social connections. They further called for more diversity in volunteering, both in terms of target groups, timing of activities and group sizes. However, they also deliberated on how diversity can be challenging.

Two of the volunteers described a need to arrange activities where individuals are matched with others in similar life situations. Thomas, a young volunteer, criticized volunteer activities for targeting mainly elderly persons and persons who are available during the daytime, ignoring the diverse groups who could need support from volunteers. He suggested that volunteers should offer more activities to young people, people who work, and students, but problematized that volunteer activities are often organized by pensioners who cannot easily relate to these life situations:

They are mainly pensioners who inhabit a totally different world than me, in a way. People who do not work. Who do not understand that someone working can also be lonely. They drive cars, go on holidays to different countries, and talk about that. And their children. They are so lucky to have children, I think. And they live a totally different life than me. It is very difficult for me to make contact with them, because I feel like a liability.

The statement suggests that being too different or being in different phases of life makes it difficult to form relationships. Another participant, Susan, also mentioned the need for broadening volunteer activities, targeting for example lonely students and individuals out of work. However, she also saw some challenges and shortcomings in activities offered to such groups of individuals. For instance, individuals out of work may have a financial situation which makes it difficult to enjoy ordinary leisure activities. Further, many of the volunteer activities targeting students are in groups and this may not fit all, as some students may also need one-to-one meetings.

Several of the other volunteers also described how individuals may have different preferences regarding whether to engage in group or one-to-one activities, as well as types of activity and that several options should be available. They had experienced that some individuals were comfortable in large groups, some prefer small groups, and others prefer one-to-one meetings. Ben said: “We are becoming a large group, and that does not fit all”. He further referred to individuals with substance use related problems and said that for those individuals, smaller groups may be more fitting as they may perhaps wish for more intimacy and privacy. This was also supported by Jill who had volunteered in a group targeting individuals in substance abuse treatment and their next of kin. They had experienced that group sizes grew and received feedback that some felt overwhelmed by relating to that many persons at once. For some, being able to take part in activities in their local community may be dependent on one-to-one contact with the volunteer, such as the person Susan visited regularly: “I think she feels it is enough to relate to only me” Susan said, and further shared that her offering to do activities with this person through one-to-one meetings seemed much appreciated. The value of one-to-one volunteer activities was also highlighted by Frank, who explained how some individuals were isolated because of disabilities or illness which makes certain types of activities, as well as activities in groups, challenging. Thomas also expressed that large groups felt strenuous to him as a volunteer: “To me, it is super-challenging to talk with ten persons at a time. It is ok with one, or two, or three, but not large groups”.

As presented above, several volunteers deliberated on how to ensure that a broader range of citizens can profit from volunteer efforts when feeling lonely or isolated. However, although they wished for volunteer efforts to target a broader range of citizens, some volunteers also described that they might struggle to interact with, and include, certain individuals in the activities they organize. Individuals with mental health problems were mentioned by some of the volunteers as a group that they might not know how to relate to, and which could pose some challenges to them. Two volunteers had experienced that meeting individuals who struggled sometimes felt burdensome, and compelled them to keep some distance, as illustrated by Susan:

Yes, so … Probably to protect myself a little bit … Because I become very involved … And I think about it a lot … And particularly if I do not get a reply in a few days, and such … I get a bit worried. Because I know that I might be one of the few persons she meets. So, I try to keep a bit distanced from it as well. Because … Yes. If not, it is quite burdensome to relate to, really … If a close friend of mine feels this way, then … .

Further, Susan said that she felt she did not know enough about mental illness and how to best communicate with individuals who experience symptoms of mental illness, and wished she had more training in this. Thus, she signalled that volunteering for this group might require taking on more professional-like roles and responsibilities. Similarly, Ben reflected upon whether activities targeting individuals with mental health or substance use problems should be arranged by the more professional volunteer organizations rather than informally arranged activities because of organizers having to take more responsibility for confidentiality within the group and making adjustments and support.

## Discussion

Our findings show that volunteers experience taking part in and facilitating a variety of relationships and roles, including friendships. These relationships are sometimes experienced as unclear, and roles may overlap and switch over time. Further, the volunteers shared examples of various ways to create spaces of trust where it felt safe for themselves and others to participate and open up to new relationships. This involved sensitivity to the emotional needs of participants, as well as making people meet and do activities of shared interest in enabling physical contexts. This indicates that the volunteers sometimes contribute to social connections for both themselves and others. Lastly, the volunteers talked about several shortcomings in the voluntary sector and called for more diversity in volunteering. However, they also deliberated how diversity can be challenging, for instance in relating to individuals with mental health problems.

The participants in our study volunteered in quite different organizations—some professional and formal, and others more personal and informal. Based on what they told about their reasons for volunteering we understand them as reflexive volunteers (Hustinx & Lammertyn, 2003), that engage in volunteer work for the social connections and well-being of both themselves and others. Some volunteers, particularly those that privately organize hiking trips open for all, described the formation of quite equal roles and reciprocal relationships—both involving themselves, as well as between others joining the hikes. We suggest that these groups where volunteers arrange activities of shared interest may be particularly valuable to foster a sense of community and what Prilleltensky (2020) calls a “we culture”. Following Prilleltensky’s (2020), these volunteers seemed to create opportunities for mattering; to feel valued and add value to self and others in the community, for instance by over time facilitating the switching of roles between receiving participant and volunteer organizer in activities. Such a sense of community and mattering is likely to increase well-being and reduce feelings of loneliness.

In some contrast, the volunteers engaged in one-to-one or group activities organized by the more formal organizations, described volunteering mainly for individuals in need of support, and thus engaging in more unequal roles such as helpers and recipients. The findings suggest they formed relationships that felt somewhere between personal and professional, and thus not fully reciprocal. In addition, some volunteers were not allowed by the organization to engage in personal relationships with participants, even though they might be invited and compelled to. These findings are in line with other studies who have shown that relationships between volunteers and individuals with health problems may be complex and be perceived as something in between therapeutic relationships and personal relationships (Cassidy et al., 2019; Mitchell & Pistrang, 2011; Ognedal et al., 2024). The parties in such relationships describe both benefits and challenges from them, and some lean on organizational support for the relationship to feel safe (Mitchell & Pistrang, 2011). Ognedal et al. (2024) found that volunteers in such relationships are required to do a lot of emotional “boundary work”, although the organization responsible for the volunteer may set formal boundaries and regulations for the relationships that evolve.

Further, our findings illustrate that volunteers may find it challenging to relate emotionally and communicate with individuals with mental health or substance use problems. These experiences are similar to other research, where community leaders expressed that individuals with mental health problems might be uncomfortable being around for volunteers and others, and may be viewed as a burden and an obstacle to volunteers’ efforts to create a sense of community in a group (Bromage et al., 2019). The volunteers in this study explained their challenges partly due to a lack knowledge about mental health difficulties and wondered if organizing activities for individuals with such problems should be more professionalized. The volunteers suggested that there should perhaps be offered courses about how to communicate with individuals with mental health challenges and wished for more organizational support.

Research indicates that support of social connections might be a necessity for some individuals with mental health or substance use problems (Sheridan et al., 2018; Vigdal et al., 2023), and both Reed et al. (2020) and Sheridan et al. (2018) argue that support provided by volunteers and others in general community arenas is sometimes key for success. Further, research indicates that volunteering for individuals with mental health problems may help reduce stigmatizing attitudes towards them (Bromage et al., 2019; Mitchell & Pistrang, 2011). Thus, we argue that recruitment and continuity of volunteer efforts for these individuals are of great importance. Based on our and other’s findings of how it may be challenging for volunteers to provide supported socialization and a sense of community for individuals with mental health problems, offering extended organizational support for these volunteers may be necessary. In light of volunteers increasingly reflexive and interest-driven motivations, this may become even more important for organizations to hold on to volunteers in the future (Hansen & Slagsvold, 2020; Hustinx & Lammertyn, 2003; Onstad et al., 2021). The importance of organizational support for individuals volunteering for persons with health problems is also supported by Ognedal et al. (2024) and Mitchell and Pistrang (2011). One way of doing this is by providing possibilities of continuous support from a neutral party within the organization, as this may be important for the feeling of safety and well-being of both volunteers and recipients of their efforts (Mitchell & Pistrang, 2011). Another may be to arrange organizational gatherings where volunteers meet to share experiences with each other and create community (Ognedal et al., 2024), as was also suggested by one of our participants. As suggested by Ognedal et al. (2024), organizations should engage in dialogue with volunteers about the ambiguous nature of volunteer relationships as well as the other challenges experienced by volunteers.

### Limitations of the study

The study is based on nine individual interviews with volunteers from three different volunteer groups in one Norwegian city. In keeping with our social constructionist position, the findings represent our interpretations of the participants’ experiences and perceptions, and there can be several alternative understandings—there is no “one truth” (Gergen, 2015). The findings are closely connected to the context in which they were developed but may apply to similar settings and contexts. Thus, to some extent, the findings can be transferable (Malterud, 2017).

## Conclusion

Volunteer work has potential of contributing to social connection and well-being, particularly through arranging social activities with little formal organization where relationships are established spontaneously and can be equal. However, more diversity in volunteer groups and activities seems desirable, where people regardless of backgrounds and challenges can meet and connect. Gathering mixed groups of people may also contribute to increased tolerance and acceptance of differences, which in time can decrease stigmatization of mental health problems and people struggling with such issues. Extended organizational support of volunteers may be necessary for recruitment and continuity of such volunteer efforts.

## Data Availability

To maintain participant anonymity, data are not made available to others.
